# A scoping review of transmission models for soil-transmitted helminth infections to underpin the development of a transmission model for *Strongyloides stercoralis*

**DOI:** 10.1017/S0031182024001392

**Published:** 2024-12

**Authors:** Mackrina Winslow, Juan Pablo Villanueva-Cabezas, Vito Colella, Patricia T. Campbell

**Affiliations:** 1 Department of Infectious Diseases, The University of Melbourne, at the Peter Doherty Institute for Infection and Immunity, Melbourne, VIC, Australia; 2 The Nossal Institute for Global Health, The University of Melbourne, Melbourne, VIC, Australia; 3 Department of Veterinary Biosciences, Faculty of Science, Melbourne Veterinary School, The University of Melbourne, Parkville, VIC, Australia

**Keywords:** *Ancylostoma*, *Ascaris*, compartmental, individual-based, low- and middle-income countries, multi-host, *Necator*, neglected tropical diseases, preventive interventions, *Trichuris*

## Abstract

Soil-transmitted helminth (STH) infections afflict people worldwide, especially in tropical and subtropical regions. *Strongyloides stercoralis* is distinctive from other STH nematodes by its complex life cycle features of autoinfection, parthenogenesis, and environmental reproduction. This scoping review aims to identify the structures, features, and techniques employed in existing STH models, emphasizing their potential application in describing *S. stercoralis* infection dynamics. A comprehensive search was conducted in the Medline, Embase, and Scopus databases for studies published until 14 June 2024. A total of 47 studies presenting a new model or novel adaptation of an existing model to human STH infection transmission were identified: only one described *S. stercoralis* transmission in humans. The identified models were predominantly deterministic and focused on the dynamics of mean worm load within hosts and the infectiousness of the environmental reservoir. One model addressed transmission in multi-host scenarios, as not all STH transmission cycles involve multiple hosts. Models were frequently used to simulate the effectiveness of mass drug administration, including drug efficacy and treatment coverage, while water, sanitation, and hygiene (WASH), health education, and vaccination were less explored. Given the limitation of individual-level data, compartmental models may be a reasonable starting point for *S. stercoralis* transmission. For a comprehensive understanding, incorporating parasite life cycle features into the model, exploring multi-host dynamics, including a diverse range of host heterogeneities, and assessing the impact of climatic factors like rainfall and land surface temperature on parasite survival in the environment may be beneficial, especially in settings where their importance is notable.

## Graphical abstract

**Figure:**
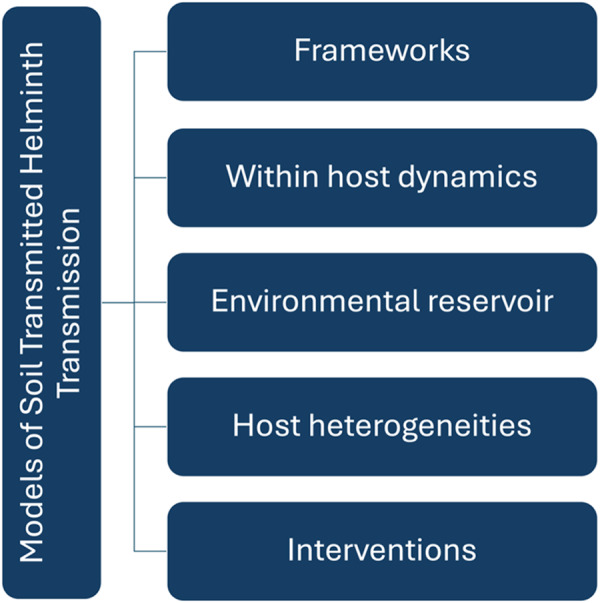

## Introduction

Soil-transmitted helminth (STH) infections afflict people worldwide, particularly those living in tropical and subtropical regions and rural areas of Sub-Saharan Africa, Latin America, China and South and Southeast Asia (Brooker *et al*., 2006; Pullan *et al*., 2014). The term STH encompasses a group of parasitic nematodes, including roundworm (*Ascaris lumbricoides*), whipworm (*Trichuris trichiura*), hookworms (*Necator americanus*, *Ancylostoma ceylanicum* and *Ancylostoma duodenale*) and *Strongyloides stercoralis* (Colella *et al*., 2021; World Health Organization, 2023). STHs are primarily transmitted through skin penetration of infective larvae or ingestion of eggs present in soil contaminated with human or animal faeces (Mbong Ngwese *et al*., 2020). In 2010, it was estimated that 1.45 billion individuals were infected with STHs, resulting in an estimated 4.98 million years lived with disability (YLDs) and 5.18 million disability-adjusted life years (DALYs) globally (Pullan *et al*., 2014).

*Strongyloides stercoralis* is distinctive from other STH nematodes due to its complex life cycle features of auto-infection, parthenogenesis and environmental reproduction (Page *et al*., 2018). Some genotyping studies (Jaleta *et al*., 2017; Nagayasu *et al*., 2017) have shown the presence of 2 distinct populations of *S. stercoralis* genotypes – one shared between dogs and humans and the other exclusive to dogs. Due to parthenogenesis and autoinfection, *S. stercoralis* infection can lead to prolonged and potentially fatal hyper infection or disseminated strongyloidiasis in infected hosts (Greiner *et al*., 2008; Toledo *et al*., 2015; Page *et al*., 2018). In 2013, it was estimated that at least 370 million individuals were infected with *S. stercoralis*, comparing the prevalence of hookworm infection and the sensitivity of diagnostic techniques for both hookworms and *S. stercoralis* (Bisoffi *et al*., 2013). However, a review study in 2017 estimated that over 600 million people were infected with *S. stercoralis* based on prevalence data collected from endemic countries worldwide between 1990 and 2016 (Buonfrate *et al*., 2020). Although *S*. *stercoralis* infection is endemic to rural areas of tropical and subtropical countries, it has also been identified in temperate regions of high-income countries, such as Australia, Japan, Spain, Italy and the United States (Krolewiecki and Nutman, 2019). Various factors are associated with the risk of this infection, including behaviour, socio-demographics, environment and historical infection exposure (Adams *et al*., 2003; Steinmann *et al*., 2007; Khieu *et al*., 2013; Fleitas *et al*., 2022). *S. fuelleborni subsp. fuelleborni* is a parasitic nematode of non-human primates that can rarely infect humans (Nutman, 2017; Al-Jawabreh *et al*., 2024). Further research is needed to clarify the distribution range of other *Strongyloides* species and the extent to which they can infect humans.

STH transmission models are primarily used to analyse the complex interplay between environmental reservoirs and hosts to understand how worm burdens and/or infection prevalence change over time. These models offer a comprehensive insight into STH transmission dynamics and are useful for forecasting trends of infection dynamics, evaluating preventive interventions and informing control programs. Components of published STH transmission models may be adapted to simulate the transmission dynamics of *S. stercoralis*, which, like other STHs, is spread through an environmental reservoir and shares some commonalities in the life cycle. Hence, this review aims to analyse published STH models to develop a new model for *S. stercoralis* transmission. Using narrative synthesis, the review identified the structures, features and techniques employed in STH models, emphasising their potential application in describing *S. stercoralis* infection dynamics. Key aspects explored include modelling the parasite's life cycle, integrating the environmental reservoir and within-host dynamics into the model, addressing host heterogeneities, including multi-host scenarios where applicable, evaluating preventive interventions and informing the choice of modelling individual worm burden or prevalence in individuals infected with *S. stercoralis*.

## Materials and methods

This scoping review was reported according to the Preferred Reporting Items for Systematic Reviews and Meta-Analyses extension for scoping reviews (PRISMA-ScR) guidelines and checklist (Tricco *et al*., 2018) (Supplementary Data S1).

### Search strategy

A comprehensive search was conducted on 14 June 2024, in the Medline, Embase and Scopus databases for studies published until 14 June 2024. The databases were searched using 2 categories of keywords, one for STH and the other for modelling, as follows:

- **STH**: sth OR helminthiasis OR helminth* OR hookworm* OR roundworm* OR whipworm* OR threadworm* OR Trichuris trichiura OR Necator americanus OR Ancylostoma ceylanicum OR Ancylostoma duodenale OR Ascaris lumbricoides OR Ascaris suum OR Strongyloides stercoralis OR ancylostomiasis OR necatoriasis OR ascariasis OR strongyloidiasis OR trichuriasis
  **AND**
- **Modelling**: mathematical model OR epidemiological model OR simulat* OR agent-based OR individual-based OR differential equation OR age-structured OR transmission model OR deterministic OR stochastic.

Medical Subject Heading (MeSH) and Embase Subject Heading (Emtree) terms were used in Medline and Embase, respectively.

### Eligibility criteria

Original peer-reviewed articles with full text available in English, presenting a modelling approach to describe the transmission dynamics of STH infections in humans, were considered eligible. Protocols, systematic or literature reviews, conference abstracts, statistical models, *in vitro* models, models that focus solely on animal infections and follow-up articles based on previously reported models that did not introduce novel modelling mechanisms were excluded. There were no restrictions on geographical areas, and the date of publication was up to 14 June 2024.

### Study selection

Two authors (PTC, MW) independently screened the title and abstract of the retrieved papers in Covidence (Veritas Health Innovation, 2023), with differences resolved through discussion between the authors. Records that met the eligibility criteria and those that required further assessment underwent full-text screening by a single author (MW) to ensure alignment with the scope and objectives of the review. The final selection of articles was determined through discussion and consensus among all authors before data extraction.

### Data extraction

An extraction tool was prepared in Microsoft Excel (version 16.75.2) to extract the following data: Study information (title, publication year, first author), study aims, considered parasites, model framework, hosts and host heterogeneities, environmental reservoir and seasonality, intervention strategies, key functions used to describe the transmission dynamics and study limitations.

### Synthesis of results

The selected studies were grouped based on several key aspects: model framework and approaches, host heterogeneities, environmental reservoir, within-host dynamics, key transmission functions and interventions. Within each group, common techniques were identified, and the differences in techniques across the studies were assessed.

As the focus of this review was to identify STH transmission model features and their potential applicability to *S. stercoralis*, a formal critical appraisal of the methodological quality of individual studies was not conducted.

## Results

**Figure 1.**
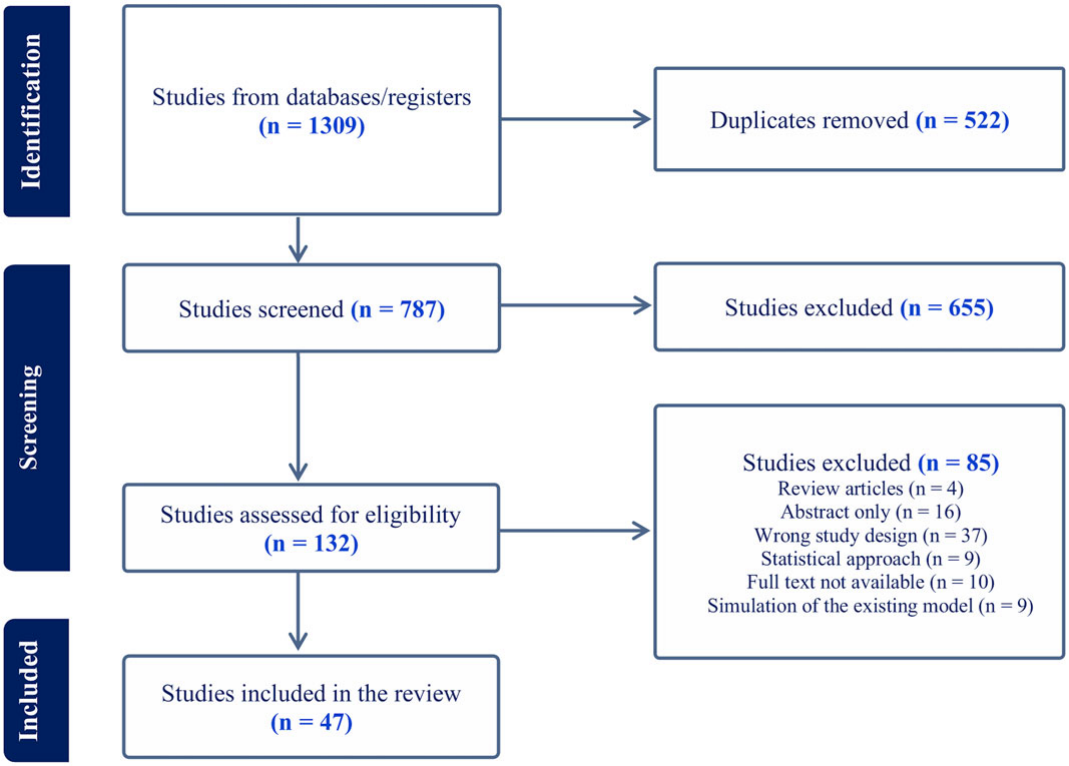
PRISMA flow diagram.

A total of 1309 articles was identified – 490 from Medline, 524 from Embase and 295 from Scopus. After deduplication, screening and full-text review, 47 papers were selected for data extraction. A PRISMA flow diagram (Fig. 1) summarizes the stages of the selection process.

### Characteristics of included studies

Table 1 presents the modelling studies in chronological order. This arrangement provides a structured overview that captures the modelling framework (compartmental or individual-based), the underlying model mechanics (deterministic or stochastic), whether the model builds upon a previous modelling effort included in this review, and the specific parasite(s) subject of investigation.

**Table 1.** Framework, approach, links to previous models, and parasites of included studies

First author (Year)	Framework	Deterministic/stochastic	Based on a model included in this study?	Parasite
Anderson and May (1982)	Differential equations for mean worm load	Deterministic	No	*A. lumbricoides*, *T. trichiura*, and hookworms (*N. americanus* and *A. duodenale*)
Anderson and May (1985b)	Differential equations for mean worm load	Deterministic	No	Helminths
Bundy *et al*. (1985)	Logistic growth equation for worm burden within a host	Deterministic	No	*T. trichiura*
Medley *et al*. (1993)	Compartmental	Deterministic and stochastic	No	*A. lumbricoides*
Chan *et al*. (1994)	Compartmental (age-structured)	Deterministic	Medley *et al*. (1993)^a^	*A. lumbricoides* and *T. trichiura*
Chan *et al*. (1997)	Compartmental	Deterministic	Medley *et al*. (1993)^b^ and Chan *et al*. (1994)^c^	Hookworms (*N. americanus* and *A. duodenale*)
Galvani (2003)	Individual-based	Stochastic	No	Helminths
Churcher *et al*. (2005)	Individual-based	Deterministic	No	*A. lumbricoides*
Walker *et al*. (2010)	Immigration-death	Stochastic	No	*A. lumbricoides*
Alexander *et al*. (2011)	Differential equations for mean worm load	Deterministic and stochastic	No	Hookworms (*N. americanus* and *A. duodenale*)
Wang *et al*. (2012)	Compartmental	Deterministic and stochastic	Medley *et al*. (1993)^d^	*A. lumbricoides* and hookworms (*N. americanus* and *A. duodenale*)
Anderson *et al*. (2013)	Compartmental	Deterministic	Chan *et al*. (1994)^c^	*A. lumbricoides*, *T. trichiura* and hookworms (*N. americanus* and *A. duodenale*)
Truscott *et al*. (2014*a*)	Compartmental (age-structured)	Deterministic and stochastic	Chan *et al*. (1994)^c^ and Anderson *et al*. (2013)^d^	*A. lumbricoides*, *T. trichiura* and hookworms (*N. americanus* and *A. duodenale*)
Truscott *et al*. (2014*b*)	Compartmental	Deterministic	No	*A. lumbricoides*, *T. trichiura* and hookworms (*N. americanus* and *A. duodenale*)
Coffeng *et al*. (2015)	Individual-based (WORMSIM)	Stochastic	No	Hookworms (*N. americanus* and *A. duodenale*)
Lo *et al*. (2015)	Compartmental (age-structured)	Deterministic	Anderson *et al*. (2013)^e^ and Chan *et al*. (1994)^e^	*A. lumbricoides*, *T. trichiura* and hookworms *(A. duodenale* and *N. americanus*)
Truscott *et al*. (2015)	Compartmental (age-structured)	Deterministic	Truscott *et al*. (2014*a*)^d^ and Truscott *et al*. (2014*b*)^d^	*A. lumbricoides*, *T. trichiura* and hookworms
Turner *et al*. (2015)	Compartmental (age-structured)	Deterministic	Truscott *et al*. (2014*b*)^d^ and Truscott *et al*. (2015)^d^	Hookworms
Bartsch *et al*. (2016)	Compartmental	Deterministic	No	Hookworms (*N. americanus*)
Medley *et al*. (2016)	Individual-based	Stochastic	Chan *et al*. (1994)^e^ and Medley *et al*. (1993)^e^	*A. lumbricoides*
Turner *et al*. (2016*b*)	Compartmental (age-structured)	Deterministic	Truscott *et al*. (2014*b*)^d^	*A. lumbricoides*
Turner *et al*. (2016*a*)	Compartmental (age-structured)	Deterministic	Truscott *et al*. (2014*b*)^d^, Truscott *et al*. (2015)^d^ and Turner *et al*. (2016*b*)^d^	*T. trichiura*
Pawelek *et al*. (2016)	Compartmental	Deterministic	No	Hookworms
Anderson *et al*. (2017)	Compartmental	Deterministic	Anderson and May (1982)^d^ and Anderson and May (1985b)^d^	*A. lumbricoides*, *T. trichiura* and hookworms (*N. americanus* and *A. duodenale*)
	Individual-based	Stochastic	No	
Farrell *et al*. (2017)	Individual-based	Stochastic	No	*A. lumbricoides*
Truscott *et al*. (2017)	Individual-based	Stochastic	Truscott *et al*. (2014*b*)^f^ and Truscott *et al*. (2015)^f^	Hookworms (*N. americanus* and *A. duodenale*)
Coffeng *et al*. (2018)	Individual-based (WORMSIM)	Stochastic	Coffeng *et al*. (2015)^g^	*A. lumbricoides*, *T. trichiura* and hookworms (*N. americanus* and *Ancyclostoma spp.*)
Davis *et al*. (2018)	Compartmental	Deterministic	No	*A. lumbricoides*
Farrell *et al*. (2018)	Differential equations for mean worm load	Deterministic	No	*A. lumbricoides*
Farrell *et al*. (2018)	Individual-based	Stochastic	Coffeng *et al*. (2015)^d^, Farrell *et al*. (2017)^d^ and Truscott *et al*. (2014*b*)^d^	*A. lumbricoides*, *T. trichiura* and hookworms (*N. americanus* and *A. duodenale*)
Werkman *et al*. (2018)	Individual-based	Stochastic	No	Hookworm (*N*. *americanus*) and *A*. *lumbricoides*
Truscott *et al*. (2019)	Individual-based	Stochastic	No	Hookworms (*N. americanus* and *A. duodenale*)
Vegvari *et al*. (2019)	Individual-based	Stochastic	Truscott *et al*. (2014*a*)^d^, Truscott *et al*. (2014*b*)^d^, Anderson *et al*. (2017)^d^ and Truscott *et al*. (2017)^d^	*A. lumbricoides* and hookworms (*N. americanus* and *A. duodenale*)
Hardwick *et al*. (2020)	Compartmental	Deterministic and Stochastic	No	*A. lumbricoides*, *T. trichiura* and hookworms (*N. americanus* and *A. duodenale*)
Lambura *et al*. (2020)	Compartmental	Deterministic	No	*A. lumbricoides*, *T. trichiura* and hookworms
Werkman *et al*. (2020)	Individual-based (age-structured)	Stochastic	Farrell *et al*. (2017)^d^	Hookworms (*N*. *americanus*) and *A*. *lumbricoides*
Malizia *et al*. (2021)	Individual-based	Stochastic	Coffeng *et al*. (2015)^d^ and Farrell *et al*. (2018)^d^	*A. lumbricoides*, *T. trichiura* and hookworms (*N. americanus* and *A. duodenale*)
Chong *et al*. (2021)	Compartmental	Deterministic	No	*A. lumbricoides*, *T. trichiura* and hookworms (*N. americanus* and *A. duodenale*)
Hardwick *et al*. (2021)	Individual-based	Stochastic	No	Hookworms (*N. americanus* and *A. duodenale*)
Truscott *et al*. (2021)	Individual-based	Stochastic	No	Hookworms (*N. americanus* and *A. duodenale*)
Okoyo *et al*. (2021)	Compartmental (age-structured)	Deterministic	Chan *et al*. (1994)^e^ and Truscott *et al*. (2014*a*)^e^	*A. lumbricoides*, *T. trichiura* and hookworms (*N. americanus* and *A. duodenale*)
Okoyo *et al*. (2022)	Compartmental (age-structured)	Deterministic	Okoyo *et al*. (2021)^h^	*A. lumbricoides*, *T. trichiura* and hookworms (*N. americanus* and *A. duodenale*)
Borlase *et al*. (2022)	Individual-based	Stochastic	Coffeng *et al*. (2015)^d^, Farrell *et al*. (2018)^d^ and Malizia *et al*. (2021)^d^	*A. lumbricoides*, *T. trichiura* and hookworms (*N. americanus* and *A. duodenale*)
Chong *et al*. (2022)	Prevalence based	Deterministic	No	*A. lumbricoides*, *T. trichiura* and hookworms (*N. americanus* and *A. duodenale*)
Walker *et al*. (2023)	Compartmental (multi-host)	Deterministic	No	*A. ceylanicum*
Oguntolu *et al*. (2024)	Compartmental	Deterministic		*A. lumbricoides*, *T. trichiura*, hookworms
Collyer and Anderson (2024)	Individual-based	Stochastic	Anderson and May (1982)^i^ and Truscott *et al*. (2014*b*)^j^	*S. stercoralis*

a The model was derived from this referenced model and expanded upon by incorporating the age heterogeneities of hosts.

b The referenced model served as the basis for describing heterogeneity in infection rates among hosts.

c The age structure of the model was built upon the framework provided by this referenced model.

d The analysis of data and assessment of the effects of various preventive strategies were conducted using this referenced model.

e The referenced model expanded by including multiple helminth infections, integrating treatment with diverse medicines, and evaluating the costs, disability, and cost-effectiveness of a mass drug administration program.

f The referenced deterministic model was used to obtain epidemiological parameters from individual level data.

g This referenced model was expanded to evaluate the impacts of WASH interventions.

h This referenced model was used to perform a sensitivity analysis.

i The referenced model was used to develop a model for studying parasite transmission and the effects of MDA.

j The referenced model was adapted to develop an individual-based stochastic model for simulating STH transmission and treatment.

### Deterministic approach

The majority of models employed a deterministic compartmental framework. Specifically, numerous models (Anderson and May, 1982, 1985*b*; Bundy *et al*., 1985; Medley *et al*., 1993; Chan *et al*., 1994, 1997; Alexander *et al*., 2011; Anderson *et al*., 2013, 2017; Truscott *et al*., 2014*a*, 2014*b*, 2015; Lo *et al*., 2015; Turner *et al*., 2015, 2016*a*, 2016*b*; Davis *et al*., 2018; Farrell and Anderson, 2018; Hardwick *et al*., 2020; Chong *et al*., 2021, 2022; Okoyo *et al*., 2021; Walker *et al*., 2023) were based on the deterministic helminth model proposed by Anderson and Anderson and May (Anderson, 1980, 1982; Anderson and May, 1985*a*, 1991) – a modelling framework that represents the parasite life cycle through 2 compartments: mature worms within the host and infective stages (eggs or larvae) in the environment. Essentially, Anderson's and Anderson & May's (Anderson, 1980, 1982; Anderson and May, 1985*a*, 1991) models employed 2 differential equations to portray the rate of change in mean worm burden within the host and *per capita* infectiousness of the environmental reservoir.

Several models based on Anderson's and Anderson & May's framework were enhanced by introducing novel elements: Medley *et al*. (1993) introduced a susceptibility factor to measure a host's relative susceptibility to parasite establishment, Chan *et al*. (1994) incorporated age structure to study different worm burdens within various age groups, Chong *et al*. (2021) introduced an impulsive mean worm model and its modified version to examine instantaneous changes in mean worm burdens in hosts immediately before and after Mass Drug Administration (MDA), Chong *et al*. (2022) developed a prevalence-based deterministic model to analyse STH transmission dynamics, focusing on prevalence rather than worm burdens, Davis *et al*. (2018) included several stages of *A. lumbricoides* worm development in their model, and Walker *et al*. (2023) developed a multi-host transmission model that includes humans and dogs.

Churcher *et al*. (2005) developed a deterministic, individual-based model estimating the number of eggs contributed by an individual host (effective transmission contribution) based on a predetermined worm burden. The model considered positive and negative density-dependent processes (Keeling and Rohani, 2011) within the parasite's life cycle. Furthermore, the authors highlighted the importance of understanding individual-level variations in worm burden and emphasized that failure to capture them may result in inaccurate estimates of transmission.

Several SEIR – susceptible, exposed, infectious and resistant (or recovered) – deterministic compartmental models have been employed to describe the transmission dynamics of parasitic infections in humans, considering various life stages of the parasite within the human and the environment. Three studies (Bartsch *et al*., 2016; Pawelek *et al*., 2016; Lambura *et al*., 2020) utilized the SEIR model structure to depict the transmission of infection among human hosts. Two studies (Bartsch *et al*., 2016; Pawelek *et al*., 2016) incorporated larval developmental stages in the environment through mutually exclusive compartments. In Bartsch *et al*. (2016), the parasite population in the environment was divided into 2 compartments: 1 for dormant eggs and non-infectious larvae and another for infectious larvae. Pawelek et al. (2016) delineated 3 distinct stages: eggs in faeces, second-stage non-infective larvae and third-stage infective larvae compartments. In contrast, the third of these SEIR models (Lambura *et al*., 2020) represented the parasite populations in the environment as a single compartment. Oguntolu *et al*. (2024) introduced a hygiene conscious (H) compartment in addition to the SEIR host compartments into their model to study the impact of hygiene awareness on infection transmission in the human population. Details of the host and parasite compartments used in the deterministic models are presented in Table 2.

**Table 2.** Compartments used in deterministic compartmental models

Compartments		Definition	Reference
Human host	Parasite		
*S*, *E*, *I*, *R*	*DL*, *IL*	Susceptible (*S*), Exposed (*E*), Infectious (*I*), Resistant (*R*), Dormant eggs and larvae that were excreted into the environment *via* infected faeces (*DL*), Infectious (L3) larvae in the environment (*IL*)	Bartsch *et al*. (2016)
*S*, *E*, *I*, *R*	*F*, *L*, *A*	Susceptible (*S*), Exposed (*E*), Infectious (*I*), Recovered individuals who were administered chemotherapy (*R*), Hookworm larva eggs in faeces (*F*), Second stage non- infective Rhabditiform larvae (*L*), Third-stage infective Filariform larvae (*A*)	Pawelek *et al*. (2016)
*S*, *E*, *I*, *R*	*M*	Susceptible (*S*), Exposed (*E*), Infectious (*I*), Recovered (*R*), Parasite population in the environment (*M*)	Lambura *et al*. (2020)
None	*S*, *M*, *W*	Mean burden of surviving worms in the population (*S*), Mean burden of worms established since treatment (*M*), Total mean parasite burden (*W* = *S* + *M*)	Medley *et al*. (1993) and Wang *et al*. (2012)
None	*M*_*i*_, *l*	Mean worm burden in the *i*th age group (*M*_*i*_), Infective larvae in the environment (*per capita* infectiousness) (*l*)	Anderson *et al*. (2017); Anderson *et al*. (2013); Chan *et al*. (1994); Chong *et al*. (2021); Hardwick *et al*. (2020); Okoyo *et al*. (2021); Okoyo *et al*. (2022); Truscott *et al*. (2014*a*); Truscott *et al*. (2014*b*); Truscott *et al*. (2015); Turner *et al*. (2016*a*); Turner *et al*. (2015) and Turner *et al*. (2016*b*)
None	*M*_*i*_	Mean worm burden in *i*th age group	Chan *et al*. (1997) and Lo *et al*. (2015)
None	*W*_*i*_	Mean number of hookworms in host *i*;*i* = humans, dogs	Walker *et al*. (2023)
None	*M*, *L*, *J*, *E*	Mean worm burden (*M*), Number of infective larval stages present in the immediate environment (*L*), Mean number of juvenile worms per host (*J*), Total count of immature eggs in the environment (*E*)	Davis *et al*. (2018)
*S*, *E*, *I*, *H*, *R*	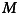	Susceptible (*S*), Exposed (*E*), Infectious (*I*), Hygiene conscious (*H*), Recovered (*R*), Parasite population in the environment (*M*)	Oguntolu *et al*. (2024)

### Stochastic approach

A subset of models (Medley *et al*., 1993, 2016; Galvani, 2003; Walker *et al*., 2010; Alexander *et al*., 2011; Wang *et al*., 2012; Truscott *et al*., 2014*a*, 2017, 2019, 2021; Coffeng *et al*., 2015; Anderson *et al*., 2017; Farrell *et al*., 2017, 2018; Coffeng *et al*., 2018; Werkman *et al*., 2018, 2020; Vegvari *et al*., 2019; Hardwick *et al*., 2020, 2021; Malizia *et al*., 2021; Borlase *et al*., 2022; Collyer and Anderson, 2024) adopted a stochastic framework. Almost all of these models employed an individual-based modelling approach that accounted for worm aggregation within the individuals, individual-level heterogeneity and tracking of individual behaviours that influence infection exposure and treatment compliance. To accommodate worm aggregation within hosts, these models depart from the assumption of uniform worm distribution by incorporating a negative binomial distribution. With this approach, it was assumed that most hosts carry a low number of worms while a small fraction carries high burdens.

The only model dedicated to *S. stercoralis* transmission, developed by Collyer and Anderson (2024), is a stochastic individual-based model that only considers human hosts. The model examined individual host behaviours and described the interactions between worm burdens within the host and larvae in the environment, capturing the randomness of infection acquisition, autoinfection, worm death and larvae maturity.

Four models (Anderson *et al*., 2017; Werkman *et al*., 2018; Vegvari *et al*., 2019; Hardwick *et al*., 2021) utilized the individual-based stochastic model described in Truscott *et al*. (2016) review paper, which focuses on modelling individual worms within hosts rather than transmission dynamics within the human population. Two of these models (Vegvari *et al*., 2019; Hardwick *et al*., 2021) expanded upon the model by integrating age structure and migration patterns into their modelling approaches.

Another 5 models (Coffeng *et al*., 2015, 2018; Farrell *et al*., 2018; Malizia *et al*., 2021; Borlase *et al*., 2022) employed the WORMSIM model framework (Coffeng *et al*., 2015), an individual-based modelling framework that facilitates the study of transmission and control of helminth infections by accounting for various individual-level heterogeneities. By design, WORMSIM calculates the infection transmission dynamics of individuals and within-host parasites stochastically, while the dynamics of infective material in the environment are simulated deterministically.

One model (Walker *et al*., 2010) employed a stochastic immigration-death process to study the acquisition of infections from the environment. This model incorporated 3 compartments of life stages of worm development within the host: pre-intestinal within-tissue migrating larval worms, ‘small’ adult worms and ‘large’ adults that develop from the small ones.

Anderson *et al*. (2017) utilized Anderson (1980)'s and Anderson and May (1982); Anderson and May (1985*b*) 's deterministic framework to predict the required MDA coverage for treating different age groups to control transmission and an individual-based stochastic framework (Truscott *et al*., 2016) to calculate the positive and negative predictive values for a defined prevalence of infection 2 years after the cessation of MDA. Similarly, many models (Medley *et al*., 1993; Wang *et al*., 2012; Hardwick *et al*., 2020) used Anderson and May's (Anderson and May, 1991) deterministic framework and extended the analysis by developing a stochastic model. Alexander *et al*. (2011) ranked the statistical power of 3 efficacy measures and used a modelling approach to estimate the likely impact of trial interventions on the force of infection.

### Host heterogeneities

The review identified several host heterogeneities that significantly influence infection transmission, including age, sex, immunity level and personal factors such as behaviour and occupation. Most models (Chan *et al*., 1994, 1997; Anderson *et al*., 2013, 2017; Truscott *et al*., 2014*a*, 2014*b*, 2015, 2017, 2019, 2021; Coffeng *et al*., 2015, 2018; Lo *et al*., 2015; Turner *et al*., 2015, 2016*a*, 2016*b*; Bartsch *et al*., 2016; Medley *et al*., 2016; Farrell *et al*., 2017, 2018; Farrell and Anderson, 2018; Werkman *et al*., 2018, 2020; Vegvari *et al*., 2019; Hardwick *et al*., 2021; Malizia *et al*., 2021; Okoyo *et al*., 2021, 2022) have incorporated age heterogeneity to account for variations in infection transmission across different age groups: pre-school aged children, school-aged children, women of childbearing age and adults. This categorization was primarily influenced by the World Health Organization's (WHO) objective of administering routine preventive chemotherapy to at least 75% of pre-school-aged children and school-aged children for STH control by 2020 (Truscott *et al*., 2016).

Individual-based frameworks, exemplified by the WORMSIM modelling framework (Coffeng *et al*., 2015, 2018), significantly contributed to understanding host-pathogen interactions by accommodating various forms of host heterogeneity. In WORMSIM, an individual's contribution to the environmental reservoir and acquisition of infection were considered to be influenced by age, sex and personal factors such as behaviour and occupation. Some studies stratified human hosts based on their immunological responsiveness (Anderson and May, 1985*b*; Galvani, 2003) and susceptibility factor (Medley *et al*., 1993; Wang *et al*., 2012). In Walker *et al*. (2010), adult worm mortality and growth rates in the host were defined as random variables to incorporate heterogeneities among hosts. In Farrell *et al*. (2017) model, individuals were assigned to a personal predisposition index to account for differential exposure to infection due to a range of possible host genetic, immunological, behavioural, social or environmental factors. Collyer and Anderson (2024)'s model included age-dependent worm acquisition from the environment, which, combined with the negative binomial distribution of new infections from the environment, resulted in heterogeneous worm burdens within the hosts.

Only one study (Walker *et al*., 2023) developed a model capable of capturing the host-species heterogeneity. Walker *et al*. (2023) incorporated both human and non-human hosts into their model and studied the interaction between different host species and the environmental reservoir. This extension sheds light on the interplay between human and animal hosts and offers a reliable framework for studying multi-host infection dynamics.

### Environmental reservoir dynamics

In considering the environmental reservoir, the *per capita* infectiousness of the shared reservoir was a key consideration, influenced by the host's contribution to the reservoir, acquisition of infection and mortality of infective larvae in the environmental reservoir. Only a few models (Galvani, 2003; Bartsch *et al*., 2016; Pawelek *et al*., 2016; Davis *et al*., 2018; Collyer and Anderson, 2024) have incorporated the developmental stages of larvae in the environment. The Collyer and Anderson (2024) model incorporated significant life stages of *S. stercoralis* in the environment: first- and second-generation larvae and mature worms. The model encompassed the environmental reproduction of *S. stercoralis* and different mortality rates among its life stages. This helps capture the parasite's persistence and worm burden in the environment, and their impact on *S. stercoralis* transmission.

In many models, the environmental reservoir was considered to be common to all age groups. To address the aspects of environmental contamination, the fecundity of worms within hosts, with or without the effect of density dependence, has been considered. Moreover, models with different host characteristics have hypothesized differences in environmental contamination and infection acquisition according to host heterogeneity.

**Figure 2.**
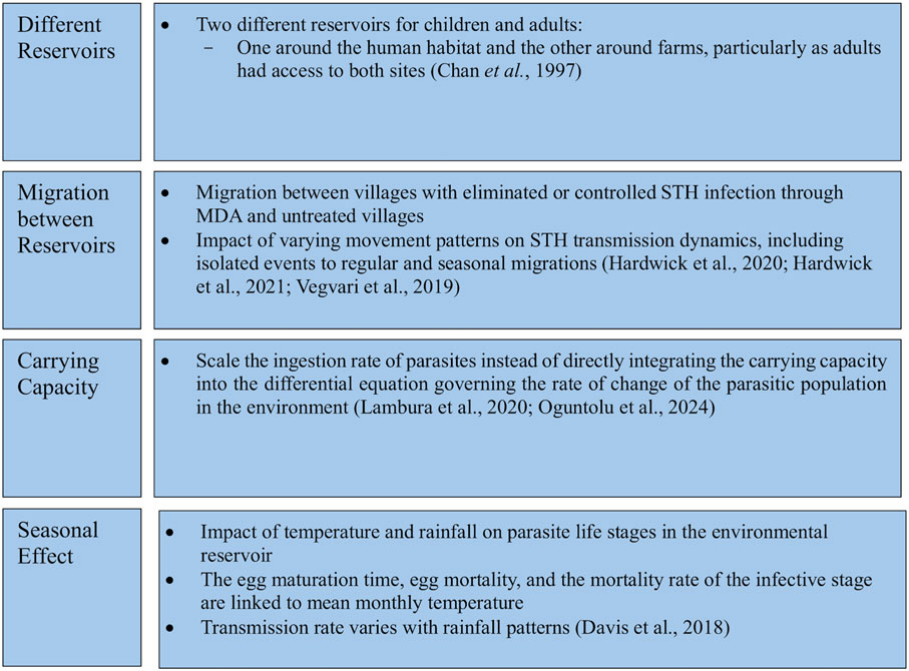
Key factors influencing environmental reservoir dynamics.

Figure 2 summarizes the factors that influence environmental dynamics, such as carrying capacity, seasonal effects and migration patterns.

### Within-host dynamics

In deterministic modelling frameworks, the primary focus was often on assessing the mean parasite burden within the hosts (Anderson and May, 1982, 1985*b*; Bundy *et al*., 1985; Medley *et al*., 1993; Chan *et al*., 1994, 1997; Alexander *et al*., 2011; Wang *et al*., 2012; Anderson *et al*., 2013; Truscott *et al*., 2014*a*, 2014*b*, 2015; Lo *et al*., 2015; Turner *et al*., 2015, 2016*a*, 2016*b*; Davis *et al*., 2018; Hardwick *et al*., 2020; Chong *et al*., 2021, 2022; Okoyo *et al*., 2021, 2022; Walker *et al*., 2023). This evaluation involved examining factors such as the strength of contact with the reservoir and the mortality rates of the hosts and worms within the host, which describes how the mean worm burden in the population changes over time.

To account for within-host dynamics, Collyer and Anderson (2024) incorporated the unique life cycle features of *S. stercoralis*, including autoinfection and reproduction dynamics, into their model. The model also simulated larvae migration from the environment, larvae maturation within the host, larvae excretion into the environment and larvae death within the host. The excretion of first-generation larvae into the environment was assumed to be proportional to the egg production within the host. Furthermore, Collyer and Anderson (2024) defined the basic reproduction number in their model as the sum of 2 terms: the basic reproduction number for free life cycle and the basic reproduction number for within host autoinfection. This approach successfully accounted for separate dynamics related to inter host and intra host *S. stercoralis* transmission.

Numerous models have integrated density-dependent fecundity of worms within hosts into their models (Anderson and May, 1982; Bundy *et al*., 1985; Medley *et al*., 1993, 2016; Chan *et al*., 1994, 1997; Galvani, 2003; Churcher *et al*., 2005; Alexander *et al*., 2011; Wang *et al*., 2012; Anderson *et al*., 2013, 2017; Truscott *et al*., 2014*a*, 2014*b*, 2015, 2019, 2021; Coffeng *et al*., 2015; Lo *et al*., 2015; Turner *et al*., 2015, 2016*a*, 2016*b*; Bartsch *et al*., 2016; Farrell *et al*., 2017; Coffeng *et al*., 2018; Davis *et al*., 2018; Farrell and Anderson, 2018; Werkman *et al*., 2018, 2020; Vegvari *et al*., 2019; Hardwick *et al*., 2020, 2021; Chong *et al*., 2021, 2022; Okoyo *et al*., 2021, 2022; Walker *et al*., 2023; Collyer and Anderson, 2024). This density-dependent fecundity captured how worm density within hosts affects their reproductive capacity and reflects the constraints imposed by the limited resources available to the worm population within the host. Additionally, the mating probability factor was incorporated to account for the impact of sexual reproduction (Bundy *et al*., 1985; Churcher *et al*., 2005; Truscott *et al*., 2014*a*, 2014*b*, 2015, 2019, 2021; Coffeng *et al*., 2015, 2018; Vegvari *et al*., 2019; Hardwick *et al*., 2020; Werkman *et al*., 2020; Chong *et al*., 2021, 2022; Malizia *et al*., 2021; Okoyo *et al*., 2021, 2022; Walker *et al*., 2023).

The WORMSIM model framework takes a more comprehensive approach by incorporating factors such as the lifespan of worms within the host, age-dependent reproductive capacity, the longevity of infective material within the host, mating cycle, male potential and female worm fecundity (Coffeng *et al*., 2015). This detailed representation aims to provide a more accurate depiction of transmission dynamics. In some models (Bundy *et al*., 1985; Walker *et al*., 2010; Bartsch *et al*., 2016; Davis *et al*., 2018) worm maturation within the host was incorporated into the model.

In most cases (Bundy *et al*., 1985; Churcher *et al*., 2005; Walker *et al*., 2010; Wang *et al*., 2012; Anderson *et al*., 2013; Truscott *et al*., 2014*b*, 2015, 2019, 2021; Coffeng *et al*., 2015; Turner *et al*., 2015, 2016*a*, 2016*b*; Bartsch *et al*., 2016; Medley *et al*., 2016; Farrell *et al*., 2017; Davis *et al*., 2018; Werkman *et al*., 2018, 2020; Hardwick *et al*., 2020; Walker *et al*., 2023), the worm burden is divided by the sex of the worms, representing a proportion of the total female worms in the host. Only a few studies (Anderson and May, 1985*b*; Medley *et al*., 1993; Galvani, 2003; Coffeng *et al*., 2015, 2018; Farrell *et al*., 2017) focused on the effect of host immunity on incoming infection and worm aggregation within the host.

### Key functions describing transmission dynamics

Some dominant parameters in STH transmission models are expressed using functional forms to depict the dynamics of transmission. The force of infection, a pivotal determinant of infection transmission dynamics, describes the rate at which hosts acquire infections through interactions with the environmental reservoir. In Walker *et al*. (2010), the force of infection is defined as the average rate that hosts acquire the adult worm. In the WORMSIM framework (Coffeng *et al*., 2015, 2018), the force of infection operates at both the population and individual levels. The population level force of infection depends on the infective material in the environment, overall exposure rate and the probability of successful infection. The individual force of infection is then a product of the population-level force of infection and individual exposure, and this individual exposure is dependent on age, sex and personal factors. Truscott *et al*. (2021), defined the force of infection for the acquisition of female worms as the product of individual strength of contact with the infectious reservoir, age-dependent contact rate and quantity of infectious material in the environmental reservoir.

Several models consider density-dependent transmission, where infection transmission depends on the density of eggs or larvae. Some models deviate from the density-dependent transmission by assuming that the population of infectious eggs or larvae in the environment is constant or at equilibrium (Walker *et al*., 2010, 2023), or by considering transmission independent of host population size (Truscott *et al*., 2021). However, several models (Chan *et al*., 1994, 1997; Hardwick *et al*., 2020; Chong *et al*., 2021, 2022) considered density-dependent transmission and then assumed the infective material in the environmental reservoir is at equilibrium for the model simulations.

For parasite reproduction, several models utilized mean egg output and mating probability of adult worms, as described by Anderson and May (1991). These parameters depend on the mean number of worms in a human population, the clumping parameter of the negative binomial distribution and density-dependent coefficient within the host. Churcher *et al*. (2005) expressed density-dependent net fecundity as a function of the number of female worms within a host, the maximum egg output per adult female worm and a measure of the severity of density-dependent fecundity. This density-dependent fecundity expression involves the exponential of the negative product of density-dependent fecundity severity measure and a number of adult female worms, indicating that as the number of adult female worms increases, fecundity experiences a rapid rise but may eventually approach a saturation point or maximum level. In WORMSIM, the reproduction capacity of a female worm is described as a product of the potential reproductive capacity of a female worm after patency, the mating factor and the exponential fecundity coefficient.

The relationship between the prevalence of infection and mean worm burden within hosts is often described by assuming a negative binomial distribution of worms per host. The functional forms used in the models to describe the force of infection, parasite reproduction and the prevalence of infection are summarized in Supplementary Table 1.

### Interventions

In modelling infection control, various interventions such as MDA, WASH (i.e. the ability to access water, sanitation and hygiene), health education initiatives and hypothetical vaccine interventions were considered.

The majority of the models focused on assessing the effectiveness of MDA programs in controlling STH infections within the communities (Anderson and May, 1982, 1985*b*; Bundy *et al*., 1985; Medley *et al*., 1993, 2016; Chan *et al*., 1994, 1997; Walker *et al*., 2010; Wang *et al*., 2012; Anderson *et al*., 2013, 2017; Truscott *et al*., 2014*a*, 2014*b*, 2015, 2017, 2021; Coffeng *et al*., 2015; Lo *et al*., 2015; Turner *et al*., 2015, 2016*a*, 2016*b*; Bartsch *et al*., 2016; Pawelek *et al*., 2016; Farrell *et al*., 2017, 2018; Coffeng *et al*., 2018; Davis *et al*., 2018; Farrell and Anderson, 2018; Werkman *et al*., 2018, 2020; Vegvari *et al*., 2019; Hardwick *et al*., 2020, 2021; Lambura *et al*., 2020; Chong *et al*., 2021; Malizia *et al*., 2021; Okoyo *et al*., 2021, 2022; Borlase *et al*., 2022; Walker *et al*., 2023; Collyer and Anderson, 2024). These models primarily evaluated the effectiveness of WHO-recommended preventive chemotherapy strategies (World Health Organization, 2013; World Health Organization, 2017). The predicted impact of several WHO-recommended drugs, including mebendazole, albendazole, ivermectin and pyrantel pamoate, was evaluated using models for different parasites, incorporating variations in efficacy, treatment frequencies and coverage levels across host strata. Furthermore, to quantify the effectiveness of MDA programs, diverse mechanisms of action were taken into account in the models: the effect of treatment on the effective reproduction number (Bundy *et al*., 1985; Anderson *et al*., 2013; Truscott *et al*., 2014*a*), reduction in mean worm burden or eggs and reduction of prevalence (Anderson and May, 1985*b*; Medley *et al*., 1993, 2016; Chan *et al*., 1994; Walker *et al*., 2010; Truscott *et al*., 2014*b*, 2015; Coffeng *et al*., 2015, 2018; Lo *et al*., 2015; Turner *et al*., 2015, 2016*a*; Bartsch *et al*., 2016; Pawelek *et al*., 2016; Farrell *et al*., 2017; Davis *et al*., 2018; Werkman *et al*., 2018, 2020; Vegvari *et al*., 2019; Chong *et al*., 2021; Hardwick *et al*., 2021; Okoyo *et al*., 2021; Collyer and Anderson, 2024). One study (Turner *et al*., 2016*b*) used the total number of worm years averted, number of years with infection and number of years with heavy infection as effectiveness metrics.

Few models incorporated WASH interventions as an STH control strategy (Coffeng *et al*., 2015, 2018; Lambura *et al*., 2020; Okoyo *et al*., 2021, 2022). Models explored different WASH modalities (e.g. none, sanitation only and hygiene only) in their capacity to modify the infectiousness of the environmental reservoir, the force of infection and changes in host contribution to the environmental reservoir. The combined impact of MDA and WASH interventions on the worm burden and the time required to interrupt STH transmission were also assessed (Coffeng *et al*., 2015, 2018; Lambura *et al*., 2020; Okoyo *et al*., 2021, 2022). In one model (Coffeng *et al*., 2015), the potential effects of health education and WASH were assumed to reduce the host's contribution to the infective material in the environmental reservoir by 50%, with equality for all hosts. Oguntolu *et al*. (2024) investigated the influence of hygiene consciousness within susceptible and infectious compartments on infection transmission.

Only 2 models (Coffeng *et al*., 2015; Lambura *et al*., 2020) incorporated the effectiveness of health education as a control measure for STH infections. In Lambura *et al*. (2020), the effectiveness measure of health education was incorporated into the force of infection, with a value of 0 indicating ineffectiveness, while a value of 1 represented complete effectiveness. Notably, Lambura *et al*. (2020) was the only study that considered all 3 preventive strategies: MDA, WASH and health education, by incorporating 3 time-dependent parameters into the model.

Walker *et al*. (2023) considered a human-only and One Health (humans and dogs) MDA strategy. They simulated different treatment scenarios for humans, including annual and biannual treatments for endemic prevalence levels of humans ≥20% and ≥50%, respectively, with 75% coverage using albendazole. Additionally, they assumed that the dogs were treated with a spot-on anthelminthic using moxidectin, with coverage ranging from 25 to 75%.

**Figure 3.**
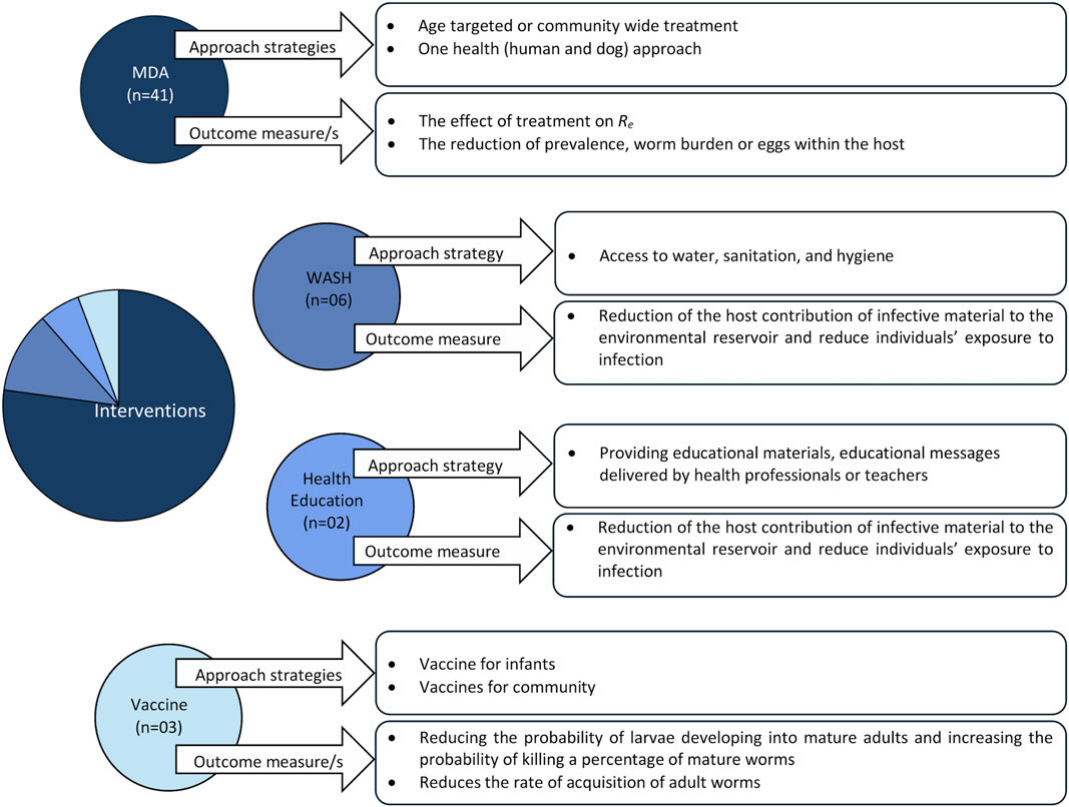
Preventive interventions and effectiveness measures.

It is worth noting that while the development of vaccination for STH infections is still an ongoing process (Zawawi and Else, 2020) and no STH vaccination is in place, some studies considered the hypothetical efficacy of vaccination as a control measure, using an arbitrary efficacy rate (Anderson and May, 1982; Alexander *et al*., 2011; Bartsch *et al*., 2016). Bartsch *et al*. (2016) modelled the vaccine to reduce the likelihood that invading L3 larvae develop into mature adults and to increase the likelihood of eliminating a proportion of mature worms present at vaccination, and Alexander *et al*. (2011) conducted an investigation to assess the statistical power of the vaccine trial interventions and their impact on reducing the force of infection. Furthermore, Anderson and May (1982) examined the proportion of the population that must be immunized with a reliable vaccine to reduce the effective reproductive number below unity. The intervention strategies and outcome measures considered in the studies are summarized in Fig. 3.

## Discussion

In this scoping review, the primary objective was to evaluate various models employed to understand the transmission dynamics of STH in order to identify techniques applicable to modelling *S. stercoralis* infection. At the time of the search, only one model describing the dynamics of *S. stercoralis* infection, focusing exclusively on humans, was identified. Researchers utilized deterministic and stochastic approaches, employing compartmental and individual-based frameworks to capture the complexity of STH transmission dynamics. Host heterogeneities, including age, sex and immunity, were often included in models. Insights into the environmental reservoir may require refinement to consider the specificities of *S. stercoralis* life stages, climatic factors on parasite survival in the environment, and the role of parasite reproduction in the environment. Understanding within-host dynamics, particularly the challenges posed by autoinfection, remains an essential focus. While interventions such as MDA, WASH, health education and vaccination were assessed using models, there is an opportunity for future modelling work to focus on a comprehensive One Health approach to reduce *S. stercoralis* transmission.

In this review, a burgeoning global interest in exploring STH transmission dynamics was identified. In the 1990s, only a few research groups delved into this area, but the review highlights a remarkable contemporary shift in research focus. This shift can be attributed to several factors, including heightened awareness of the global burden of STH infections, advances in modelling techniques, improved data availability and the acknowledgment of STH as a significant public health issue, as outlined in the WHO Roadmap for Neglected Tropical Diseases (World Health Organization, 2022).

Most models relied on a deterministic approach, possibly influenced by factors such as simplicity, the consistency of outcomes and computational efficiency. However, deterministic models may not fully capture the complexities of STH infection transmission as they overlook inherent randomness in biological processes, including worm acquisition, worm reproduction, egg or larvae deposition and parasite death. On the other hand, stochastic models offer a more realistic representation of the complexities observed in real-world infection transmission scenarios and are well suited for capturing randomness in biological processes. This approach allows for running multiple simulations capturing many possible futures, enabling the analysis of the distribution of possible outcomes and the estimation of probabilities.

The choice of the framework to describe the transmission dynamics of *S. stercoralis* infection should align with the research objectives, considerations and data availability. Compartmental models, describing average behaviour in homogeneous compartments (host/parasite), prove valuable for certain investigations. Individual-based models are particularly suitable for studying transmission dynamics within individuals, especially in scenarios with low prevalence or with important host heterogeneities. Due to the limitations of individual-level data and to reduce the complexities associated with the inherent randomness in the biology of *S. stercoralis*, deterministic compartmental models may serve as a reasonable starting point for modelling *S. stercoralis* transmission.

The prevalence of *S. stercoralis* is impacted by age, sex and various behavioural factors, exhibiting distinct patterns compared to other STH nematodes (Khieu et al., 2014). One of the main findings in this review is that less attention has been given to incorporating host heterogeneities into STH models. The majority of models focused on age disparities in their model simulations, often with a primary focus on child-targeted prevention programs. However, Coffeng *et al*. (2018) considered the importance of incorporating host diversities such as age, sex and personal factors into the model in controlling the infection.

Moreover, understanding the role of multiple hosts in pathogen transmission may be important to capture *S. stercoralis* transmission dynamics and the impact of control strategies. Walker *et al*. (2023) supported this by incorporating humans and dogs into the *A. ceylanicum* transmission model and highlighting the importance of One Health interventions. Collyer and Anderson (2024) examined *S. stercoralis* infection transmission solely in humans while acknowledging uncertainties regarding the involvement of dogs as hosts in *S. stercoralis* transmission. However, several studies have found *S. stercoralis* cross infection between humans and dogs (Bradbury and Streit, 2024) and the existence of a lineage of *S. stercoralis* that can infect humans and dogs (Jaleta *et al*., 2017; Nagayasu *et al*., 2017). Hence, dogs' contribution to the reservoir and their acquisition of *S. stercoralis* can influence the infection dynamics of the environmental reservoir and, subsequently, the dynamics of *S. stercoralis* transmission in humans. Moreover, the behaviour of dogs, including roaming patterns and defecation, might influence the spatial distribution of larvae in the environment, thereby affecting the risk of human exposure. A model incorporating both hosts, humans and dogs, would allow exploration of the contribution of dogs to the human burden of strongyloidiasis by capturing the interconnection between hosts that occurs *via* the environment. A greater understanding of these transmission processes could facilitate the design of an effective control strategy to reduce the overall burden of infection.

In modelling the environmental reservoir – a factor that plays a significant role in the STH parasite life cycle dynamics – most models have considered the contribution of hosts to the environmental reservoir, the acquisition of infective material from the reservoir and the survival of infective material in the environment. However, there has been a tendency to overlook the seasonal effects on the environmental reservoir (only included in Davis *et al*. (2018)) and the carrying capacity of larvae or eggs in the environment (Lambura *et al*., 2020; Oguntolu *et al*., 2024). *S. stercoralis* exhibits unique features in its free-living cycle, involving parasite reproduction in the environment and the short life expectancy of infective larvae, distinguishing it from other STHs. The Collyer and Anderson (2024) model comprehensively captured these *S. stercoralis* life cycle stages and underscored their importance of considering parasite's life cycle in modelling to understanding transmission dynamics. Furthermore, modelling of *S. stercoralis* may requires special attention to the environmental risk factors such as annual rainfall, land surface temperature and land coverage (Forrer *et al*., 2019).

Within-host dynamics were frequently focused on mean worm loads, larvae maturation and reproduction within the host. Parameters influencing transmission between the host and the reservoir and the life span of the worm in the host contributed to describing the mean worm burden. Additionally, incorporating fecundity and mating probability into the model addressed parasite reproduction within the host in some of the reviewed STH models. In modelling *S. stercoralis* within host dynamics, Collyer and Anderson (2024) incorporated *S. stercoralis* life cycle features, including autoinfection and within-host reproduction, and successfully captured the resulting worm burdens within the host. This also exemplified the importance of life cycle features on transmission dynamics.

Interventions, mainly MDA, were extensively modelled for human populations. Since *S. stercoralis* epidemiology is interrelated with humans, dogs and the environment (Page *et al*., 2018; Bradbury and Streit, 2024), achieving optimal outcomes may necessitate interventions that target infection control across these 3 sectors. Designing interventions to control the parasite population in the environment directly may be less critical due to *S. stercoralis*' free-living cycle being limited to a single, short-lived and generation (Page *et al*., 2018). However, it remains crucial to manage parasite shedding from hosts into the environment and reduce host exposure to infective larvae, as this is the primary route of *S. stercoralis* transmission for humans and dogs. While the potential role of dogs in transmitting *S. stercoralis* remains uncertain, experimental studies have shown that *S. stercoralis* can infect both humans and dogs (Bradbury and Streit, 2024). Moreover, several genotyping studies have identified different genotypes of *S. stercoralis*, with one lineage capable of causing infections in both species and another exclusively in dogs (Jaleta *et al*., 2017; Nagayasu *et al*., 2017). Future population genomic studies will help elucidate the transmission dynamics of *S. stercoralis* between dogs and humans. Hence, models may be used to demonstrate whether dogs play a significant role in *S. stercoralis* transmission through environmental contamination, acquisition and the spread of infections. If this the case, considering dogs in intervention strategies may provide an additional avenue to control *S. stercoralis* infection in humans. Future modelling efforts can incorporate interventions targeting humans and/or dogs to estimate the likely impact of different control strategies on disease burden.

The one reason for the literature gap in modelling the dynamics of *S. stercoralis* infection in humans, dogs and the environment is the uncertainties associated with the biology of the parasite. Moreover, a notable limitation identified in this review is the scarcity of data for parameterization of STH models. This data limitation introduces parameter uncertainty, potentially resulting in significant disparities between model predictions and actual data. Furthermore, consideration of fixed age groups, and less attention to environmental reservoir were mainly acknowledged.

This scoping review provides a comprehensive overview of the existing literature to identify the structures and mechanisms that describe STH transmission dynamics and critical gaps in the current literature on STH infection transmission modelling, particularly with *S. stercoralis* infection. With this focus, the review did not critically appraise each article to consider whether it was ‘fit-for-purpose’. Some modelling studies described in review articles (Anderson *et al*., 2014; Truscott *et al*., 2016) and book chapters (Anderson and May, 1991) were not included in the study, and hence, some unique features may have been missed relevant to *S. stercoralis*, although we consider this unlikely.

## Conclusion

Control of *S. stercoralis* infection has been complicated by diagnostic challenges. However, WHO has recognized the significance of controlling *S. stercoralis* infection, incorporating preventive chemotherapy strategies into their publication ‘Ending the Neglect to Attain the Sustainable Development Goals: A Road Map for neglected tropical diseases 2021–2030’. This map underscores the need to investigate the transmission dynamics and to develop effective control methods for *S. stercoralis* infection, as they can positively impact human and animal health.

The review identified several key insights: different methods for modelling interaction dynamics between hosts and the environment, with a focus on infection acquisition and transmission mechanisms; modelling parasite life cycle dynamics within hosts and the external environment, encompassing processes such as reproduction and maturation; exploring multi-host dynamics; and inclusion of a diverse range of host heterogeneities in models. Furthermore, approaches to incorporating seasonal effects on the environmental reservoir into the model and analysing preventive interventions were also identified. These findings, particularly the mathematical techniques used to incorporate the parasite's life cycle features into the model, offer valuable guidance for understanding and controlling *S. stercoralis* transmission. A remaining gap for the development of *S. stercoralis* models is parameter estimation, given the many uncertainties related to diagnostic sensitivities and limitations on the knowledge of basic *S. stercoralis* biology. Future models for *S. stercoralis* would benefit from exploration of parameter space that encompasses these uncertainties.

## Supporting information

Winslow et al. supplementary material Winslow et al. supplementary material

## Data Availability

The data supporting the findings of this study are available within the article and its supplementary materials.

## Supplementary material

The supplementary material for this article can be found at https://doi.org/10.1017/S0031182024001392.

## References

[ref1] Adams M, Page W and Speare R (2003) Strongyloidiasis: an issue in Aboriginal communities. Rural and Remote Health 3, 152. 15877491

[ref3] Alexander N, Cundill B, Sabatelli L, Bethony JM, Diemert D, Hotez P, Smith PG, Rodrigues LC and Brooker S (2011) Selection and quantification of infection endpoints for trials of vaccines against intestinal helminths. Vaccine 29, 3686–3694. 21435404 10.1016/j.vaccine.2011.03.026 PMC3093614

[ref2] Al-Jawabreh R, Anderson R, Atkinson LE, Bickford-Smith J, Bradbury RS, Breloer M, Bryant AS, Buonfrate D, Cadd LC, Crooks B, Deiana M, Grant W, Hallem E, Hedtke SM, Hunt V, Khieu V, Kikuchi T, Kounosu A, Lastik D, Van Lieshout L, Liu Y, Mcsorley HJ, Mcveigh P, Mousley A, Murcott B, Nevin WD, Noskova E, Pomari E, Reynolds K, Ross K, Streit A, Suleiman M, Tiberti N and Viney M (2024) Strongyloides questions-a research agenda for the future. Philosophical Transactions of the Royal Society B 379, 20230004. 10.1098/rstb.2023.0004 PMC10676812 38008122

[ref4] Anderson RM (1980) The dynamics and control of direct life cycle helminth parasites. Vito Volterra Symposium on Mathematical Models in Biology: Proceedings of a Conference Held at the Centro Linceo Interdisciplinare, Accademia Nazionale dei Lincei, Rome December 17–21, 1979.

[ref5] Anderson, RM (1982) The population dynamics and control of hookworm and roundworm infections. In The Population Dynamics of Infectious Diseases: Theory and Applications. Springer, pp. 67–108. doi: 10.1007/978-1-4899-2901-3_3.

[ref6] Anderson RM and May RM (1982) Population dynamics of human helminth infections: control by chemotherapy. Nature 297, 557–563. 7088139 10.1038/297557a0

[ref7] Anderson RM and May RM (1985 *a*) Helminth infections of humans: mathematical models, population dynamics, and control. Advances in Parasitology 24, 1–101. 3904343 10.1016/s0065-308x(08)60561-8

[ref8] Anderson RM and May RM (1985 *b*) Herd immunity to helminth infection and implications for parasite control. Nature 315, 493–496. 4000277 10.1038/315493a0

[ref9] Anderson RM and May RM (1991) Infectious Diseases of Humans: Dynamics and Control. Oxford University Press.

[ref10] Anderson RM, Truscott JE, Pullan RL, Brooker SJ and Hollingsworth TD (2013) How effective is school-based deworming for the community-wide control of soil-transmitted helminths? PLoS Neglected Tropical Diseases 7, e2027. 23469293 10.1371/journal.pntd.0002027 PMC3585037

[ref11] Anderson R, Truscott J and Hollingsworth TD (2014) The coverage and frequency of mass drug administration required to eliminate persistent transmission of soil-transmitted helminths. Philosophical Transactions of the Royal Society of London. Series B, Biological Sciences 369, 20130435. 24821921 10.1098/rstb.2013.0435 PMC4024228

[ref12] Anderson R, Farrell S, Turner H, Walson J, Donnelly CA and Truscott J (2017) Assessing the interruption of the transmission of human helminths with mass drug administration alone: optimizing the design of cluster randomized trials. Parasites & Vectors 10, 93. 28212667 10.1186/s13071-017-1979-x PMC5316156

[ref13] Bartsch SM, Hotez PJ, Hertenstein DL, Diemert DJ, Zapf KM, Bottazzi ME, Bethony JM, Brown ST and Lee BY (2016) Modeling the economic and epidemiologic impact of hookworm vaccine and mass drug administration (MDA) in Brazil, a high transmission setting. Vaccine 34, 2197–2206. 27002501 10.1016/j.vaccine.2016.03.018 PMC5547742

[ref14] Bisoffi Z, Buonfrate D, Montresor A, Requena-Méndez A, Muñoz J, Krolewiecki AJ, Gotuzzo E, Mena MA, Chiodini PL, Anselmi M, Moreira J and Albonico M (2013) *Strongyloides stercoralis*: a plea for action. PLoS Neglected Tropical Diseases 7, e2214. 23675546 10.1371/journal.pntd.0002214 PMC3649953

[ref15] Borlase A, Le Rutte EA, Castaño S, Blok DJ, Toor J, Giardina F and Davis EL (2022) Evaluating and mitigating the potential indirect effect of COVID-19 on control programmes for seven neglected tropical diseases: a modelling study. The Lancet. Global Health 10, e1600–e1611. 36240827 10.1016/S2214-109X(22)00360-6 PMC9579354

[ref16] Bradbury RS and Streit A (2024) Is strongyloidiasis a zoonosis from dogs? Philosophical Transactions of the Royal Society of London. Series B, Biological Sciences 379, 20220445. 38008118 10.1098/rstb.2022.0445 PMC10676807

[ref17] Brooker S, Clements AC and Bundy DA (2006) Global epidemiology, ecology and control of soil-transmitted helminth infections. Advances in Parasitology 62, 221–261. 16647972 10.1016/S0065-308X(05)62007-6 PMC1976253

[ref18] Bundy DA, Thompson DE, Cooper ES, Golden MH and Anderson RM (1985) Population dynamics and chemotherapeutic control of *Trichuris trichiura* infection of children in Jamaica and St. Lucia. Transactions of the Royal Society of Tropical Medicine and Hygiene 79, 759–764. 3832488 10.1016/0035-9203(85)90110-5

[ref19] Buonfrate D, Bisanzio D, Giorli G, Odermatt P, Fürst T, Greenaway C, French M, Reithinger R, Gobbi F, Montresor A and Bisoffi Z (2020) The global prevalence of *Strongyloides stercoralis* infection. Pathogens 9, 468. 32545787 10.3390/pathogens9060468 PMC7349647

[ref20] Chan MS, Guyatt HL, Bundy DA and Medley GF (1994) The development and validation of an age-structured model for the evaluation of disease control strategies for intestinal helminths. Parasitology 109(Pt 3), 389–396. 7970893 10.1017/s0031182000078422

[ref21] Chan MS, Bradley M and Bundy DA (1997) Transmission patterns and the epidemiology of hookworm infection. International Journal of Epidemiology 26, 1392–1400. 9447422 10.1093/ije/26.6.1392

[ref22] Chong NS, Smith SR, Werkman M and Anderson RM (2021) Modelling the ability of mass drug administration to interrupt soil-transmitted helminth transmission: community-based deworming in Kenya as a case study. PLoS Neglected Tropical Diseases 15, e0009625. 34339450 10.1371/journal.pntd.0009625 PMC8360579

[ref23] Chong NS, Hardwick RJ, Smith SR, Truscott JE and Anderson RM (2022) A prevalence-based transmission model for the study of the epidemiology and control of soil-transmitted helminthiasis. PLoS One 17, e0272600. 36006929 10.1371/journal.pone.0272600 PMC9409602

[ref24] Churcher TS, Ferguson NM and Basáñez MG (2005) Density dependence and overdispersion in the transmission of helminth parasites. Parasitology 131(Pt 1), 121–132. 16038403 10.1017/s0031182005007341

[ref25] Coffeng LE, Bakker R, Montresor A and De Vlas SJ (2015) Feasibility of controlling hookworm infection through preventive chemotherapy: a simulation study using the individual-based WORMSIM modelling framework. Parasites & Vectors 8, 541. 26489659 10.1186/s13071-015-1151-4 PMC4618856

[ref26] Coffeng LE, Vaz Nery S, Gray DJ, Bakker R, De Vlas SJ and Clements AC (2018) Predicted short and long-term impact of deworming and water, hygiene, and sanitation on transmission of soil-transmitted helminths. PLoS Neglected Tropical Diseases 12, e0006758. 30522129 10.1371/journal.pntd.0006758 PMC6283645

[ref27] Colella V, Khieu V, Worsley A, Senevirathna D, Muth S, Huy R, Odermatt P and Traub RJ (2021) Risk profiling and efficacy of albendazole against the hookworms *Necator americanus* and *Ancylostoma ceylanicum* in Cambodia to support control programs in Southeast Asia and the Western Pacific. The Lancet Regional Health. Western Pacific 16. doi: 10.1016/j.lanwpc.2021.100258 . PMC8403762 34590062

[ref28] Collyer BS and Anderson R (2024) The transmission dynamics of *Strongyloides stercoralis* and the impact of mass drug administration. Philosophical Transactions of the Royal Society of London. Series B, Biological Sciences 379, 20220442. 38008114 10.1098/rstb.2022.0442 PMC10676814

[ref29] Davis EL, Danon L, Prada JM, Gunawardena SA, Truscott JE, Vlaminck J, Anderson RM, Levecke B, Morgan ER and Hollingsworth TD (2018) Seasonally timed treatment programs for *Ascaris lumbricoides* to increase impact – an investigation using mathematical models. PLoS Neglected Tropical Diseases 12, e0006195. 29346383 10.1371/journal.pntd.0006195 PMC5773001

[ref30] Farrell SH and Anderson RM (2018) Helminth lifespan interacts with non-compliance in reducing the effectiveness of anthelmintic treatment. Parasites & Vectors 11, 66. 29382359 10.1186/s13071-018-2670-6 PMC5791166

[ref31] Farrell SH, Truscott JE and Anderson RM (2017) The importance of patient compliance in repeated rounds of mass drug administration (MDA) for the elimination of intestinal helminth transmission. Parasites & Vectors 10, 291. 28606164 10.1186/s13071-017-2206-5 PMC5469187

[ref32] Farrell SH, Coffeng LE, Truscott JE, Werkman M, Toor J, De Vlas SJ and Anderson RM (2018) Investigating the effectiveness of current and modified World Health Organization guidelines for the control of soil-transmitted helminth infections. Clinical Infectious Diseases: An Official Publication of the Infectious Diseases Society of America 66(suppl_4), S253–S259. 29860285 10.1093/cid/ciy002 PMC5982801

[ref33] Fleitas PE, Kehl SD, Lopez W, Travacio M, Nieves E, Gil JF, Cimino RO and Krolewiecki AJ (2022) Mapping the global distribution of *Strongyloides stercoralis* and hookworms by ecological niche modeling. Parasites & Vectors 15, 197. 35676740 10.1186/s13071-022-05284-w PMC9178904

[ref34] Forrer A, Khieu V, Vounatsou P, Sithithaworn P, Ruantip S, Huy R, Muth S and Odermatt P (2019) *Strongyloides stercoralis*: spatial distribution of a highly prevalent and ubiquitous soil-transmitted helminth in Cambodia. PLoS Neglected Tropical Diseases 13, e0006943. 31220075 10.1371/journal.pntd.0006943 PMC6586258

[ref35] Galvani AP (2003) Immunity, antigenic heterogeneity, and aggregation of helminth parasites. Journal of Parasitology 89, 232–241. 12760634 10.1645/0022-3395(2003)089[0232:IAHAAO]2.0.CO;2

[ref36] Greiner K, Bettencourt J and Semolic C (2008) Strongyloidiasis: a review and update by case example. American Society for Clinical Laboratory Science 21, 82–88. 18507302

[ref37] Hardwick RJ, Vegvari C, Truscott JE and Anderson RM (2020) The ‘breakpoint’ of soil-transmitted helminths with infected human migration. Journal of Theoretical Biology 486, 110076. 31733259 10.1016/j.jtbi.2019.110076 PMC6977101

[ref38] Hardwick RJ, Werkman M, Truscott JE and Anderson RM (2021) Stochastic challenges to interrupting helminth transmission. Epidemics 34, 100435. 33571786 10.1016/j.epidem.2021.100435

[ref39] Jaleta TG, Zhou S, Bemm FM, Schär F, Khieu V, Muth S, Odermatt P, Lok JB and Streit A (2017) Different but overlapping populations of *Strongyloides stercoralis* in dogs and humans – dogs as a possible source for zoonotic strongyloidiasis. PLoS Neglected Tropical Diseases 11, e0005752. 28793306 10.1371/journal.pntd.0005752 PMC5565190

[ref40] Keeling MJ and Rohani P (2011) Modeling Infectious Diseases in Humans and Animals. Princeton University Press. doi: 10.2307/j.ctvcm4gk0.5.

[ref41] Khieu V, Schär F, Marti H, Sayasone S, Duong S, Muth S and Odermatt P (2013) Diagnosis, treatment and risk factors of *Strongyloides stercoralis* in schoolchildren in Cambodia. PLoS Neglected Tropical Diseases 7, e2035. 23409200 10.1371/journal.pntd.0002035 PMC3566990

[ref42] Khieu V, Schär F, Marti H, Bless PJ, Char MC, Muth S and Odermatt P (2014) Prevalence and risk factors of *Strongyloides stercoralis* in Takeo Province, Cambodia. Parasites & Vectors 7, 221. 24886763 10.1186/1756-3305-7-221 PMC4029906

[ref43] Krolewiecki A and Nutman TB (2019) Strongyloidiasis: a neglected tropical disease (NTD). Infectious Disease Clinics of North America 33, 135–151. 30712758 10.1016/j.idc.2018.10.006 PMC6367705

[ref44] Lambura AG, Mwanga GG, Luboobi L and Kuznetsov D (2020) Mathematical model for optimal control of soil-transmitted helminth infection. Computational and Mathematical Methods in Medicine 2020, 6721919. 32802152 10.1155/2020/6721919 PMC7416292

[ref45] Lo NC, Bogoch II, Blackburn BG, Raso G, N'goran EK, Coulibaly JT, Becker SL, Abrams HB, Utzinger J and Andrews JR (2015) Comparison of community-wide, integrated mass drug administration strategies for schistosomiasis and soil-transmitted helminthiasis: a cost-effectiveness modelling study. The Lancet. Global Health 3, e629–e638. 26385302 10.1016/S2214-109X(15)00047-9

[ref46] Malizia V, Giardina F, Vegvari C, Bajaj S, Mcrae-Mckee K, Anderson RM, De Vlas SJ and Coffeng LE (2021) Modelling the impact of COVID-19-related control programme interruptions on progress towards the WHO 2030 target for soil-transmitted helminths. Transactions of the Royal Society of Tropical Medicine and Hygiene 115, 253–260. 33313897 10.1093/trstmh/traa156 PMC7798673

[ref47] Mbong Ngwese M, Prince Manouana G, Nguema Moure PA, Ramharter M, Esen M and Adégnika AA (2020) Diagnostic techniques of soil-transmitted helminths: impact on control measures. Tropical Medicine and Infectious Disease 5, 93. 32516900 10.3390/tropicalmed5020093 PMC7344795

[ref48] Medley GF, Guyatt HL and Bundy DAP (1993) A quantitative framework for evaluating the effect of community treatment on the morbidity due to ascariasis. Parasitology 106(Pt 2), 211–221. 8446474 10.1017/s0031182000075016

[ref49] Medley GF, Turner HC, Baggaley RF, Holland C and Hollingsworth TD (2016) The role of more sensitive helminth diagnostics in mass drug administration campaigns: elimination and health impacts. Advances in Parasitology 94, 343–392. 27756457 10.1016/bs.apar.2016.08.005

[ref50] Nagayasu E, Aung M, Hortiwakul T, Hino A, Tanaka T, Higashiarakawa M, Olia A, Taniguchi T, Win SMT, Ohashi I, Odongo-Aginya EI, Aye KM, Mon M, Win KK, Ota K, Torisu Y, Panthuwong S, Kimura E, Palacpac NMQ, Kikuchi T, Hirata T, Torisu S, Hisaeda H, Horii T, Fujita J, Htike WW and Maruyama H (2017) A possible origin population of pathogenic intestinal nematodes, *Strongyloides stercoralis*, unveiled by molecular phylogeny. Scientific Reports 7, 4844. 28687738 10.1038/s41598-017-05049-x PMC5501853

[ref51] Nutman TB (2017) Human infection with *Strongyloides stercoralis* and other related *Strongyloides* species. Parasitology 144, 263–273. 27181117 10.1017/S0031182016000834 PMC5563389

[ref52] Oguntolu FA, Peter OJ, Yusuf A, Omede B, Bolarin G and Ayoola T (2024) Mathematical model and analysis of the soil-transmitted helminth infections with optimal control. Modeling Earth Systems and Environment 10, 883–897.

[ref53] Okoyo C, Medley G, Mwandawiro C and Onyango N (2021) Modeling the interruption of the transmission of soil-transmitted helminths infections in Kenya: modeling deworming, water, and sanitation impacts. Frontiers in Public Health 9, 637866. 33842421 10.3389/fpubh.2021.637866 PMC8024473

[ref54] Okoyo C, Onyango N, Orowe I, Mwandawiro C and Medley G (2022) Sensitivity analysis of a transmission interruption model for the soil-transmitted helminth infections in Kenya. Frontiers in Public Health 10, 841883. 35400031 10.3389/fpubh.2022.841883 PMC8990131

[ref55] Page W, Judd JA and Bradbury RS (2018) The unique life cycle of *Strongyloides stercoralis* and implications for public health action. Tropical Medicine and Infectious Disease 3, 53. 30274449 10.3390/tropicalmed3020053 PMC6073624

[ref56] Pawelek KA, Liu S and Lolla MU (2016) Modeling the spread of hookworm disease and assessing chemotherapy programs: mathematical analysis and comparison with surveillance data. Journal of Biological Systems 24, 167–191.

[ref57] Pullan RL, Smith JL, Jasrasaria R and Brooker SJ (2014) Global numbers of infection and disease burden of soil transmitted helminth infections in 2010. Parasites & Vectors 7, 37. 24447578 10.1186/1756-3305-7-37 PMC3905661

[ref58] Steinmann P, Zhou XN, Du ZW, Jiang JY, Wang LB, Wang XZ, Li LH, Marti H and Utzinger J (2007) Occurrence of *Strongyloides stercoralis* in Yunnan Province, China, and comparison of diagnostic methods. PLoS Neglected Tropical Diseases 1, e75. 17989788 10.1371/journal.pntd.0000075 PMC2041812

[ref59] Toledo R, Munoz-Antoli C and Esteban JG (2015) Strongyloidiasis with emphasis on human infections and its different clinical forms. Advances in Parasitology 88, 165–241. 25911368 10.1016/bs.apar.2015.02.005

[ref60] Tricco AC, Lillie E, Zarin W, O'brien KK, Colquhoun H, Levac D, Moher D, Peters MDJ, Horsley T, Weeks L, Hempel S, Akl EA, Chang C, Mcgowan J, Stewart L, Hartling L, Aldcroft A, Wilson MG, Garritty C, Lewin S, Godfrey CM, Macdonald MT, Langlois EV, Soares-Weiser K, Moriarty J, Clifford T, Tunçalp Ö and Straus SE (2018) PRISMA extension for scoping reviews (PRISMA-ScR): checklist and explanation. Annals of Internal Medicine 169, 467–473. 30178033 10.7326/M18-0850

[ref61] Truscott JE, Hollingsworth TD and Anderson R (2014 *a*) Modeling the interruption of the transmission of soil-transmitted helminths by repeated mass chemotherapy of school-age children. PLoS Neglected Tropical Diseases 8, e3323. 25474477 10.1371/journal.pntd.0003323 PMC4256169

[ref62] Truscott JE, Hollingsworth TD, Brooker SJ and Anderson RM (2014 *b*) Can chemotherapy alone eliminate the transmission of soil transmitted helminths? Parasites & Vectors 7, 266. 24916278 10.1186/1756-3305-7-266 PMC4079919

[ref63] Truscott JE, Turner HC and Anderson RM (2015) What impact will the achievement of the current World Health Organisation targets for anthelmintic treatment coverage in children have on the intensity of soil transmitted helminth infections? Parasites & Vectors 8, 551. 26490544 10.1186/s13071-015-1135-4 PMC4618937

[ref64] Truscott JE, Turner HC, Farrell SH and Anderson RM (2016) Soil-transmitted helminths: mathematical models of transmission, the impact of mass drug administration and transmission elimination criteria. Advances in Parasitology 94, 133–198. 27756454 10.1016/bs.apar.2016.08.002

[ref65] Truscott JE, Werkman M, Wright JE, Farrell SH, Sarkar R, Ásbjörnsdóttir K and Anderson RM (2017) Identifying optimal threshold statistics for elimination of hookworm using a stochastic simulation model. Parasites & Vectors 10, 321. 28666452 10.1186/s13071-017-2256-8 PMC5493114

[ref66] Truscott JE, Ower AK, Werkman M, Halliday K, Oswald WE, Gichuki PM, Mcharo C, Brooker S, Njenga SM, Mwandariwo C, Walson JL, Pullan R and Anderson R (2019) Heterogeneity in transmission parameters of hookworm infection within the baseline data from the TUMIKIA study in Kenya. Parasites & Vectors 12, 442. 31522687 10.1186/s13071-019-3686-2 PMC6745791

[ref67] Truscott JE, Hardwick RJ, Werkman M, Saravanakumar PK, Manuel M, Ajjampur SSR, Ásbjörnsdóttir KH, Khumbo K, Witek-Mcmanus S, Simwanza J, Cottrell G, Houngbégnon P, Ibikounlé M, Walson JL and Anderson RM (2021) Forecasting the effectiveness of the DeWorm3 trial in interrupting the transmission of soil-transmitted helminths in three study sites in Benin, India and Malawi. Parasites & Vectors 14, 67. 33472677 10.1186/s13071-020-04572-7 PMC7818558

[ref68] Turner HC, Truscott JE, Bettis AA, Shuford KV, Dunn JC, Hollingsworth TD, Brooker SJ and Anderson RM (2015) An economic evaluation of expanding hookworm control strategies to target the whole community. Parasites & Vectors 8, 570. 26542226 10.1186/s13071-015-1187-5 PMC4635541

[ref69] Turner HC, Truscott JE, Bettis AA, Hollingsworth TD, Brooker SJ and Anderson RM (2016 *a*) Analysis of the population-level impact of co-administering ivermectin with albendazole or mebendazole for the control and elimination of *Trichuris trichiura*. Parasite Epidemiology and Control 1, 177–187. 27430028 10.1016/j.parepi.2016.02.004 PMC4946157

[ref70] Turner HC, Truscott JE, Fleming FM, Hollingsworth TD, Brooker SJ and Anderson RM (2016 *b*) Cost-effectiveness of scaling up mass drug administration for the control of soil-transmitted helminths: a comparison of cost function and constant costs analyses. The Lancet. Infectious Diseases 16, 838–846. 26897109 10.1016/S1473-3099(15)00268-6

[ref71] Vegvari C, Truscott JE, Kura K and Anderson RM (2019) Human population movement can impede the elimination of soil-transmitted helminth transmission in regions with heterogeneity in mass drug administration coverage and transmission potential between villages: a metapopulation analysis. Parasites & Vectors 12, 438. 31522681 10.1186/s13071-019-3612-7 PMC6745807

[ref72] Veritas Health Innovations (2023) Covidence Systematic Review Software. Melbourne, Australia: Veritas Health Innovations. Available at www.covidence.org.

[ref73] Walker M, Hall A and Basáñez MG (2010) Trickle or clumped infection process? A stochastic model for the infection process of the parasitic roundworm of humans, *Ascaris lumbricoides*. International Journal for Parasitology 40, 1381–1388. 20620142 10.1016/j.ijpara.2010.07.001

[ref74] Walker M, Lambert S, Neves MI, Worsley AD, Traub R and Colella V (2023) Modeling the effectiveness of One Health interventions against the zoonotic hookworm *Ancylostoma ceylanicum*. Frontiers in Medicine 10, 1092030. 36960338 10.3389/fmed.2023.1092030 PMC10028197

[ref75] Wang J, Li HZ, Chen YD, Liu CH and Tang LH (2012) Chemotherapy-based control of ascariasis and hookworm in highly endemic areas of China: field observations and a modeling analysis. Biomedical and Environmental Sciences 25, 272–281. 22840577 10.3967/0895-3988.2012.03.004

[ref76] Werkman M, Toor J, Vegvari C, Wright JE, Truscott JE, Ásbjörnsdóttir KH, Rubin Means A, Walson JL and Anderson RM (2018) Defining stopping criteria for ending randomized clinical trials that investigate the interruption of transmission of soil-transmitted helminths employing mass drug administration. PLoS Neglected Tropical Diseases 12, e0006864. 30273343 10.1371/journal.pntd.0006864 PMC6181437

[ref77] Werkman M, Wright JE, Truscott JE, Oswald WE, Halliday KE, Papaiakovou M, Farrell SH, Pullan RL and Anderson RM (2020) The impact of community-wide, mass drug administration on aggregation of soil-transmitted helminth infection in human host populations. Parasites & Vectors 13, 290. 32513254 10.1186/s13071-020-04149-4 PMC7278197

[ref78] World Health Organization (2013) Sustaining the Drive to Overcome the Global Impact of Neglected Tropical Diseases. Geneva, Switzerland: World Health Organization.

[ref79] World Health Organization (2017) Guideline: Preventive Chemotherapy to Control Soil-Transmitted Helminth Infections in at-Risk Population Groups. Geneva, Switzerland: World Health Organization. 29578660

[ref80] World Health Organization (2022) Ending the Neglect to Attain the Sustainable Development Goals: A Rationale for Continued Investment in Tackling Neglected Tropical Diseases 2021–2030. Geneva, Switzerland: World Health Organization.

[ref81] World Health Organization (2023) Soil-Transmitted Helminth Infections. World Health Organization. Retrieved 10 May 2023 from https://www.who.int/news-room/fact-sheets/detail/soil-transmitted-helminth-infections.

[ref82] Zawawi A and Else KJ (2020) Soil-transmitted helminth vaccines: are we getting closer? Frontiers in Immunology 11, 576748. 33133094 10.3389/fimmu.2020.576748 PMC7565266

