# Supplementary material for: A scoping review of transmission models for soil-transmitted helminth infections to underpin the development of a transmission model for Strongyloides stercoralis
Source: Parasitology. 2024 Nov 15;151(14):1508–21. doi: 10.1017/S0031182024001392 (PMC12052426; doi:10.1017/S0031182024001392)
Supplement: Winslow et al. supplementary material [file S0031182024001392sup001.docx]

**Appendix. Supplementary material**

**Supplementary Data S1**. Preferred Reporting Items for Systematic reviews and Meta-Analyses extension for Scoping Reviews (PRISMA-ScR) Checklist

| **SECTION** | **ITEM** | **PRISMA-ScR CHECKLIST ITEM** | **REPORTED ON PAGE #** |
| --- | --- | --- | --- |
| **TITLE** | | | |
| Title | 1 | Identify the report as a scoping review. | 1 |
| **ABSTRACT** | | | |
| Structured summary | 2 | Provide a structured summary that includes (as applicable): background, objectives, eligibility criteria, sources of evidence, charting methods, results, and conclusions that relate to the review questions and objectives. | 2 |
| **INTRODUCTION** | | | |
| Rationale | 3 | Describe the rationale for the review in the context of what is already known. Explain why the review questions/objectives lend themselves to a scoping review approach. | 4,5 |
| Objectives | 4 | Provide an explicit statement of the questions and objectives being addressed with reference to their key elements (e.g., population or participants, concepts, and context) or other relevant key elements used to conceptualise the review questions and/or objectives. | 5 |
| **METHODS** | | | |
| Protocol and registration | 5 | Indicate whether a review protocol exists; state if and where it can be accessed (e.g., a Web address); and if available, provide registration information, including the registration number. | 5 |
| Eligibility criteria | 6 | Specify characteristics of the sources of evidence used as eligibility criteria (e.g., years considered, language, and publication status), and provide a rationale. | 6 |
| Information sources* | 7 | Describe all information sources in the search (e.g., databases with dates of coverage and contact with authors to identify additional sources), as well as the date the most recent search was executed. | 6 |
| Search | 8 | Present the full electronic search strategy for at least 1 database, including any limits used, such that it could be repeated. | 6 |
| Selection of sources of evidence† | 9 | State the process for selecting sources of evidence (i.e., screening and eligibility) included in the scoping review. | 7 |
| Data charting process‡ | 10 | Describe the methods of charting data from the included sources of evidence (e.g., calibrated forms or forms that have been tested by the team before their use, and whether data charting was done independently or in duplicate) and any processes for obtaining and confirming data from investigators. | 7 |
| Data items | 11 | List and define all variables for which data were sought and any assumptions and simplifications made. | 7 |
| Critical appraisal of individual sources of evidence§ | 12 | If done, provide a rationale for conducting a critical appraisal of included sources of evidence; describe the methods used and how this information was used in any data synthesis (if appropriate). | - |
| Synthesis of results | 13 | Describe the methods of handling and summarising the data that were charted. | 7 |
| **RESULTS** | | | |
| Selection of sources of evidence | 14 | Give numbers of sources of evidence screened, assessed for eligibility, and included in the review, with reasons for exclusions at each stage, ideally using a flow diagram. | Figure 1 |
| Characteristics of sources of evidence | 15 | For each source of evidence, present characteristics for which data were charted and provide the citations. | 8 |
| Critical appraisal within sources of evidence | 16 | If done, present data on critical appraisal of included sources of evidence (see item 12). | - |
| Results of individual sources of evidence | 17 | For each included source of evidence, present the relevant data that were charted that relate to the review questions and objectives. | 8-21, Table 1, Table 2, Figure 2, Figure 3, Supplementary Table |
| Synthesis of results | 18 | Summarise and/or present the charting results as they relate to the review questions and objectives. | Table 1, Table 2, Figure 2, Figure 3, Supplementary Table |
| **DISCUSSION** | | | |
| Summary of evidence | 19 | Summaries the main results (including an overview of concepts, themes, and types of evidence available), link to the review questions and objectives, and consider the relevance to key groups. | 21 |
| Limitations | 20 | Discuss the limitations of the scoping review process. | 25,26 |
| Conclusions | 21 | Provide a general interpretation of the results with respect to the review questions and objectives, as well as potential implications and/or next steps. | 26,27 |
| **FUNDING** | | | |
| Funding | 22 | Describe sources of funding for the included sources of evidence, as well as sources of funding for the scoping review. Describe the role of the funders of the scoping review. | 27 |

JBI = Joanna Briggs Institute; PRISMA-ScR = Preferred Reporting Items for Systematic reviews and Meta-Analyses extension for Scoping Reviews.

* Where *sources of evidence* (see second footnote) are compiled from, such as bibliographic databases, social media platforms, and Web sites.

† A more inclusive/heterogeneous term used to account for the different types of evidence or data sources (e.g., quantitative and/or qualitative research, expert opinion, and policy documents) that may be eligible in a scoping review as opposed to only studies. This is not to be confused with *information sources* (see first footnote).

‡ The frameworks by Arksey and O'Malley (6) and Levac and colleagues (7) and the JBI guidance (4, 5) refer to the process of data extraction in a scoping review as data charting*.*

§ The process of systematically examining research evidence to assess its validity, results, and relevance before using it to inform a decision. This term is used for items 12 and 19 instead of "risk of bias" (which is more applicable to systematic reviews of interventions) to include and acknowledge the various sources of evidence that may be used in a scoping review (e.g., quantitative and/or qualitative research, expert opinion, and policy document).

**Supplementary Table:** Functional forms for the force of infection, parasite reproduction within the hosts and the prevalence of infection.

| **Parameter** | **Description** | **Assumptions** | **References** |
| --- | --- | --- | --- |
| Force of infection ($\lambda(t)$) | $\lambda=m_{c}\varphi$  $m_{c}-$ Mean number of eggs containing female larvae acquired per infectious contact,  $\varphi-$Rate of infectious contacts with clumps of eggs in the environment. | - The environmental reservoir contains *A. lumbricoides* eggs - The number of *A. lumbricoides* eggs in the environment is constant and at a temporal equilibrium - Frequency dependent transmission | (Walker *et al.*, 2010) |
|  | The overall force of infection acting on the human population in month 't',  $lr\left( t \right)=\bar{lu}\left( t \right)\cdot\zeta\cdot v$  $\bar{lu}\left( t \right)-$Infective material in the reservoir $\zeta-$Scalar representing the overall exposure rate  $v -$Probability that an infective particle in the reservoir successfully matures into a stage capable of infecting humans  Then, the force of infection acting upon individual $i$ of age $a$ and sex $s$is,  $foi_{i}\left( t \right)=lr\left( t \right).\frac{{Ex}_{i}}{\sum_{i=1}^{N(t)} {Ex}_{i}}$  $lr\left( t \right)-$Overall force of infection acting on the human population  ${Ex}_{i} -$ Relative exposure of an individual accounting for their age, sex, and personal factors | - The environmental reservoir contains eggs and larvae - Infective material in the environmental reservoir decay exponentially - Density-dependent transmission | (Coffeng *et al.*, 2015; Coffeng *et al.*, 2018) |
|  | $\lambda=\frac{\beta M}{\kappa+M}$  $\beta-$ Intake rate of eggs/larvae  $\kappa-$Density of the helminths at which the infection rate is half the maximum rate  $M-$ Parasite population in the environment | - The environmental reservoir is the soil containing the parasitic worms - Infective material in the environmental reservoir decays naturally at a constant rate - Transmission depends on the density of infective material until $M=\kappa$ | ([Lambura *et al.*, 2020](#_ENREF_43); [Oguntolu *et al.*, 2024](#_ENREF_50)) |
|  | $\lambda=\beta A$  $\beta-$ Infection rate  $A-$ Number of filariform larvae; third-stage infective larvae | - The environmental reservoir is the soil containing the hookworm larvae - Infective material in the environmental reservoir decays naturally at a constant rate - Density dependent transmission | ([Pawelek et al., 2016](#_ENREF_52)) |
|  | $F_{i}\left( a,t \right)=\eta_{i}{\beta(a}_{i})L(t)$  $F_{i}\left( a,t \right)-$ Force of infection for the acquisition of female worms in individual $i$  $\eta_{i} -$ Individual strength of contact with the infectious reservoir  ${\beta(a}_{i}) -$ Age-dependent contact rate  $L\left( t \right) -$ Quantity of infectious material in the environmental reservoir | - The environmental reservoir contains infectious eggs and larvae - Density-dependent transmission | ([Truscott et al., 2021](#_ENREF_57)) |
| Parasite Reproduction | $f\left( M \right)=\left[ 1+\frac{\left( 1-z \right)M}{k} \right]^{-(k+1)}$  $f\left( M \right)-$Mean egg output per gram of stool  $M -$Mean number of worms in a human population of size N at time t  $k -$Clumping parameter of the negative binomial distribution  $\gamma-$ Strength of density-dependent constraints  $z= e^{-\gamma}$ | - Worms distributed in a negative binomial pattern - Density dependent fecundity is considered | ([Alexander et al., 2011](#_ENREF_2); [Anderson et al., 1982](#_ENREF_7); [Anderson et al., 2013](#_ENREF_11); [Bartsch et al., 2016](#_ENREF_12); [Chan et al., 1994](#_ENREF_19); [Chong et al., 2022](#_ENREF_20); [Chong et al., 2021](#_ENREF_21); [Hardwick et al., 2020](#_ENREF_35); [Lo et al., 2015](#_ENREF_43); [Medley et al., 1993](#_ENREF_46); [Okoyo et al., 2021](#_ENREF_49); [Okoyo et al., 2022](#_ENREF_50); [Truscott et al., 2021](#_ENREF_57); [Truscott et al., 2014a](#_ENREF_58); [Truscott et al., 2014b](#_ENREF_59); [Truscott et al., 2019](#_ENREF_60); [Truscott et al., 2015](#_ENREF_61); [Walker et al., 2023](#_ENREF_70); [Wang et al., 2012](#_ENREF_71)) |
|  | $\emptyset\left( M \right)=1-\left[ \frac{1+\frac{\left( 1-z \right)M}{k}}{1+\frac{(2-z)M}{2k}} \right]^{k+1}$  $\emptyset\left( M \right) -$ Mating probability of adult worms  $M -$Mean number of worms in a human population of size N at time t  $k -$Clumping parameter of the negative binomial distribution  $\gamma-$Strength of density-dependent constraints  $z= e^{-\gamma}$ | - Worms distributed in a negative binomial pattern - Worms are polygamous - All female worms mated if at least one male worm is present | ([Chong et al., 2022](#_ENREF_20); [Chong et al., 2021](#_ENREF_21); [Hardwick et al., 2020](#_ENREF_35); [Okoyo et al., 2021](#_ENREF_49); [Okoyo et al., 2022](#_ENREF_50); [Truscott et al., 2021](#_ENREF_57); [Truscott et al., 2014a](#_ENREF_58); [Truscott et al., 2014b](#_ENREF_59); [Truscott et al., 2019](#_ENREF_60); [Truscott et al., 2015](#_ENREF_61); [Walker et al., 2023](#_ENREF_70)) |
|  | The mean number of eggs per gram of feces produced,  $\Theta_{A}=\frac{\lambda_{0}}{C_{F}}\left[ 1-e^{(\frac{-C_{F}w}{2})} \right]$  $w -$ Mean number of worms per host  $\lambda_{0}-$ Maximum number of eggs produced per gram of faeces per adult female worm  $C_{F}-$Measure of the severity of density-dependent fecundity | - Used for directly transmitting worms - Assumed $1:1$ sex ratio of worms withing the host - Density dependent fecundity is considered | ([Churcher et al., 2005](#_ENREF_22)) |
|  | The reproductive capacity of a patent female worm of age $a$at time $t$is,  $r\left( a,t \right)=R\left( a-pp \right).m\left( t \right).z(t)$  $R\left( a-pp \right)-$ Potential reproductive capacity of a female worm after patency  $pp -$Prepatent age  $m\left( t \right) -$ Mating factor at time $t$  $z\left( t \right) -$ The exponential fecundity coefficient at time $t$  $z\left( t \right)=e^{-w(t)\lambda_{z}}$  $w\left( t \right)-$ Number of adult worms  $\lambda_{z} -$ Amount of negative density dependence | - The lifespan of worms within the host is random variable - Female worms produce eggs after prepatent age - Female worms must inseminate in each reproductive cycle to produce eggs | ([Coffeng et al., 2015](#_ENREF_23); [Coffeng et al., 2018](#_ENREF_24)) |
| The prevalence of infection ($P$) | $P=1-\left( 1+\frac{M}{k} \right)^{-k}$  $M-$ Mean number of worms in a human population  $k-$ Clumping parameter of the negative binomial distribution | - Negative binomial distribution of worms per host | ([Anderson et al., 2017](#_ENREF_3); [Anderson et al., 1982](#_ENREF_7); [Anderson et al., 2013](#_ENREF_11); [Bartsch et al., 2016](#_ENREF_12); [Bundy et al., 1985](#_ENREF_16); [Chong et al., 2022](#_ENREF_20); [Chong et al., 2021](#_ENREF_21); [Lo et al., 2015](#_ENREF_43); [Walker et al., 2023](#_ENREF_70); [Werkman et al., 2018](#_ENREF_72)) |
